# Extrusion Conditions and Amylose Content Affect Physicochemical Properties of Extrudates Obtained from Brown Rice Grains

**DOI:** 10.1155/2013/584148

**Published:** 2013-05-23

**Authors:** Rolando José González, Elena Pastor Cavada, Javier Vioque Peña, Roberto Luis Torres, Dardo Mario De Greef, Silvina Rosa Drago

**Affiliations:** ^1^Instituto de Tecnología de Alimentos, Universidad Nacional del Litoral, 1 de Mayo 3250, 3000 Santa Fe, Argentina; ^2^Instituto de la Grasa (CSIC), Avenida Padre García Tejero 4, 41012 Seville, Spain; ^3^Consejo Nacional de Investigaciones Científicas y Técnicas (CONICET), Avenida Rivadavia 1917, 1033 Ciudad Autónoma de Buenos Aires, Argentina

## Abstract

The utilization of whole grains in food formulations is nowadays recommended. Extrusion cooking allows obtaining precooked cereal products and a wide range of ready-to-eat foods. Two rice varieties having different amylose content (Fortuna 16% and Paso 144, 27%) were extruded using a Brabender single screw extruder. Factorial experimental design was used to study the effects of extrusion temperature (160, 175, and 190°C) and grits moisture content (14%, 16.5%, and 19%) on extrudate properties. Specific mechanical energy consumption (SMEC), radial expansion (*E*), specific volume (SV), water absorption (WA), and solubility (*S*) were determined on each extrudate sample. In general, Fortuna variety showed higher values of SMEC and *S* (703–409 versus 637–407 J/g; 33.0–21.0 versus 20.1–11.0%, resp.) than those of Paso 144; on the contrary SV (8.64–3.47 versus 8.27–4.53 mL/g) and WA tended to be lower (7.7–5.1 versus 8.4–6.6 mL/g). Both varieties showed similar values of expansion rate (3.60–2.18). Physical characteristics depended on extrusion conditions and rice variety used. The degree of cooking reached by Paso rice samples was lower than that obtained for Fortuna. It is suggested that the presence of germ and bran interfered with the cooking process, decreasing friction level and broadening residence time distribution.

## 1. Introduction

The utilization of whole grains in food formulations is nowadays much recommended. The beneficial effects of including whole grains in the diet have been demonstrated by several authors [1]. Whole grains are rich in nutritive, functional, and phytochemical compounds [2].

Cereal food manufactures have responded to these advantages with the development of new whole grains and rich fiber products, most of them being in the form of flakes or blends such as muslin. Beside that, the demand of precooked cereal products, in the form of snacks, breakfast cereals, and flours, has increased during the last decades. Market globalization and changes on food consumption model of developing countries have been the main factors for this increasing demand [3].

The most used processes for the production of precooked cereal foods are flaking, puffing, and extrusion [4]. Extrusion cooking is considered not only an HTST process, but also a versatile one to obtain a wide range of ready-to-eat cereal foods. HTST process is recommended when keeping the nutritional value is important.

Among cereals, rice is one of the three most important. It is considered a staple food for more than 3,000 million people and provides them with about 60% of their daily energy intake [5]. The unique properties of rice, such as its hypoallergenicity, bland taste, white color, and easy digestion, make it a very desirable grain for new food development [6–9]. Rice is most consumed as grain in its three traditional forms: white, parboiled, and brown rice. 

It is well known that much of the vitamins and minerals are lost during the polishing step of white rice processing. Even though protein content of rice is lower than that in maize and wheat, it is richer in lysine [10]. On the other hand, an increase of demand of nongluten products based on rice has been verified in the last decades [11], and there is a need of novel formulas in order to diversify the use of rice other than the traditional one.

Amylose content affects quality and texture of cooked rice [12, 13]. Rice varieties containing higher amylose content are normally more resistant to hydration [14] and give more viscous flour dispersion during water cooking [15]. Amylose content affecting the texture of gluten-free bread is controversial. It has been reported that flour from rice containing less than 20% amylose is desirable to use in bread without gluten formulas since it produces much less retrogradation [16], but other authors [7] have shown that the use of rice varieties, having medium and high amylose content (18–27%), gave good results for gluten-free bread. Moreover, Juliano [17] pointed out that waxy rice is preferred for cakes and pudding, because its flour is easy to hydrate and gives to the products better stability. On the other hand, high amylose rice variety is recommended for the production of noodles and is preferred for the production of expanded snacks products [15, 18, 19].

The advantages of extrusion as a cooking process are well known and have been discussed by several authors [20, 21]. The wide range of degree of cooking of starchy materials that can be obtained by extrusion is remarkable, since it is possible to produce samples with low degree of cooking (including loss of crystallinity and granule destruction) to fully cooked samples (highly destructed granules and total loss of native crystallinity) [18, 22, 23]. Water dispersion prepared with precooked flour obtained by extrusion under condition of high degree of cooking has much less viscosity than dispersion prepared with flour precooked by other processes, which can be considered a nutritional advantage [3]. 

The effects of extrusion variables on structural changes and product properties of starchy materials have been extensively studied by several authors [23–27]and particularly for rice [6–8, 19, 28–32]. However, studies regarding the processing effect and product properties from brown rice having different amylose content are scarce.

In the present work, two commercial rice varieties with different amylose content were selected to analyze the effect of extrusion variables on energy consumption and whole grains extrudate properties, using surface response methodology.

## 2. Materials and Methods

Two commercial long rice varieties, Paso 144 (28% amylose) and Fortuna (16% amylose), with length/width ratio of 3.5 and 2.3, respectively, were provided by Molino Trimacer (Santa Fe-Argentina) in the form of dehulled (or brown) grain. Brown rice was milled to obtain grits with a particle size between 1.190 and 0.420 mm, using a Buhler Miag, roll mill, according to a milling diagram [24, 33]. Moisture content, crude protein, fat content (expressed as petroleum ether extract), and ash content were determined by AOAC methods [34].

The extrusion process was carried out with a Brabender 20 DN single screw extruder, using a 4 : 1 compression ratio screw, a 3/20 mm (diameter/length) die, and a screw speed of 150 rpm. The effects of grits moisture (*M*) and extrusion temperature (*T*) were analyzed by surface response methodology using a factorial design 3^2^ with triplicate of the central point, resulting in 11 runs for each rice variety. The levels of each variable were as follows: *T*: 160–175–190°C and *M*: 14–16.5–19%. Rice grits samples were conditioned by adding water to reach the moisture level corresponding to each experimental sample, 1 h before each run.

Each extruded sample was obtained as soon as the stationary condition was reached, with torque and mass output simultaneously measured. These values were used to determine the specific mechanical energy consumption (SMEC) [23, 24] using the following formula: SMEC (J g^−1^) = *k* · *T* · *N* · *Qa* ^−1^, where *k* is: 61.6 10^−3^, *T* is torque in Brabender units (BU), *N* is screw rpm, and *Qa* (g/min) is the mass output, referring to feeding moisture level. The *k* value takes into account unit conversion and constants. All extruded samples were air-dried in an oven at 50°C until a moisture content of 6% was reached, this moisture level being considered adequate for texture evaluation. Each dried sample was divided into several portions and kept in plastic bags hermetically sealed until their evaluation. Diameters were measured with a Vernier caliper on ten pieces of sample, and axial expansion (*E*) was determined as the ratio *E* = *D* · *d* ^−1^, where *D* is the extrudate diameter (average of ten determinations) and *d* is the die diameter. Extrudate specific volume (SV) was obtained by calculating the volume/weight (d · b) ratio (as cm^3^/g), corresponding to an extrudate piece of about 15 cm long. This procedure was applied to ten pieces and the average is reported. 

Water solubility and water absorption were determined according to González et al. [23]. An amount (150 g) representative of each extrudate sample was first ground with a laboratory hammer mill (Retsch-Muhle-Germany) using a 2 mm sieve and then with a Cyclotec mill (UD Corp Boulder Colorado-USA) using a 1 mm sieve. 

Water solubility was done by dispersing 2.5 g of flour in 50 mL water, agitating during 30 min. and centrifuging at 2000 g; soluble solids were obtained after evaporation in an oven at 105°C and calculated as soluble solids 100 g of flour (d · b). Water absorption was determined using Bauman method and expressed as mL of water/g of sample. 

One extruded sample from each rice variety was selected to evaluate flour dispersion viscosity, and rheograms were carried out for two solids concentrations (8 and 11%, W/W), at 60°C, using an RV3 Haake Rotovisco viscometer (Germany). In every case, power law parameters *k* and *n* (*τ* = *kG* ^*n*^) were estimated by regression. 

### 2.1. Statistical Analysis

The average of duplicate of each determination is reported. Analysis of Variance was carried out using the software Statgraphics Plus 3.0, and the statistical differences among samples were determined using the LSD test.

## 3. Results and Discussion

Table 1 shows the composition of the raw materials. The values are similar to those reported in the literature for brown rice [35].

Table 2 shows results of specific mechanical energy consumption (SMEC), expansion, specific volume (SV), water absorption (WA), and solubility (*S*%) corresponding to the eleven experimental extruded samples and for the two rice varieties. 

Table 3 shows the degree of significance (*P* value), corresponding to each term of the regression model obtained for each response. The lack of fit was not significant (*P* > 0.5), except for WA corresponding to Paso 144. 

It is observed that, for all responses, one or more terms of the regression model were significant (*P* < 0.05). Linear terms *M* and *T* were significant in all cases, except for *M* for WA corresponding to Paso 144 (*P* < 0.6779) and *T* for solubility corresponding to both rice varieties (*P* < 0.2544 for *F* and *P* < 0.1337 for *P*). Quadratic terms (*M* ^2^ and *T* ^2^) were significant only in the following cases: (a) *M* ^2^ in expansion and WA for Fortuna variety and in expansion for Paso 144 variety (b) *T* ^2^ in SV for Fortuna and also in *S*% for Paso 144.

### 3.1. Specific Mechanical Energy Consumption

**Figure 1:**
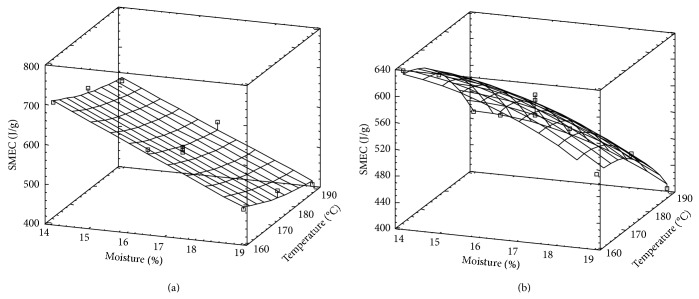
Surface response for SMEC corresponding to Fortuna rice variety (a) and Paso 144 rice variety (b).

SMEC surface response corresponding to each rice variety is observed in Figures 1(a) and 1(b). 

The surface corresponding to Fortuna is planar, but that of Paso 144 shows a curvature in the *T* direction. According to the ANOVA results (Table 2), only the linear terms (*H* and *T*) were significant (*P* < 0.05), but, in the case of Paso 144, the degree of significance of the term *T* ^2^ is not negligible (*P* < 0.097) and that explains the curvature in *T* direction. In both cases, SMEC was inversely related to both *H* and *T*, since as *H* and *T* increased, friction level in the extruder decreased and consequently SMEC decreased [23, 36]. Several authors working with others materials, such as wheat, quinoa, or barley, have found similar effect [32, 37–39]. 

SMEC values of both varieties were in similar range, although those corresponding to Fortuna tend to be slightly higher, particularly at low *M* level, which is in agreement with the results of water solubility (Table 2) which is discussed later on this paper. Since starch fraction of Fortuna rice variety reached higher degree of cooking during extrusion (higher *S*), energy dissipation (SMEC) would be also higher than that in the case of higher amylose content. Moreover, the presence of fiber and amylose content would reduce SMEC, since when low and high amylose polished rice varieties are extruded, higher level of solubility was obtained [22].

### 3.2. Radial Expansion

**Figure 2:**
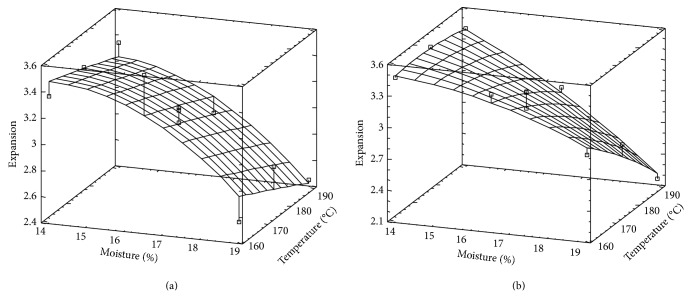
Surface response for expansion corresponding to Fortuna rice variety (a) and Paso 144 rice variety (b).

Radial expansion values of both varieties were in a similar range. Expansion response surfaces corresponding to each rice variety are observed in Figures 2(a) and 2(b). In the case of Fortuna, the curvature in *M* direction is explained by the significance of the term *M* ^2^ (*P* < 0.0448). While in the case of Paso 144, the expected plane is modified by the influence of the term *M* × *T*, whose significance is not negligible (0.0614). 

Both varieties show similar tendency as that of SMEC, supporting the idea that expansion rate is directly related to the degree of friction inside the extruder [23, 40]. As it is expected, expansion values corresponding to both varieties are higher at the lower level of *M*, since this extrudate property is positively correlated with the elastic component of the melt coming out from the die, which would decrease as *T* or *M* increases [3]. This inverse relationship between *E* and *M* was also found by Hagenimana et al. [6] and Pedrosa Silva Clérici and El-Dash [7]. Regarding *T* effect, Hagenimana et al. [6] and Singh et al. [32] have shown that a maximum of *E* exists at intermediate *T* (130–150°C). However, this temperature range is lower than that we used.

### 3.3. Specific Volume

Specific Volume has been proposed as a good indicator of degree of cooking (DC) for cereal extruded products, because DC is directly related to granule structure destruction [23].

**Figure 3:**
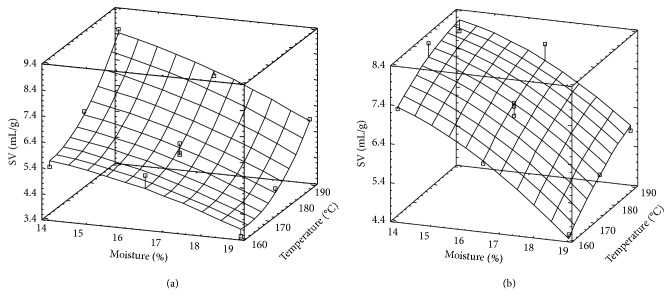
Surface response for specific volume (SV) corresponding to Fortuna rice variety (a) and Paso 144 rice variety (b).

In most of the cases, SV values corresponding to Paso were slightly higher than those of Fortuna. Response surfaces corresponding to each rice variety are observed in Figures 3(a) and 3(b). In the case of Fortuna, the curvature in *T* direction is explained by the significance of the term *T* ^2^ (*P* < 0.0415), which also can explain the high dependence of this response on temperature. In the case of Paso 144, the expected planar surface is modified by the term *M* ^2^ whose significance is not too low (*P* < 0.0968).

The effects of variables followed similar tendency for both varieties. SV decreases as *M* increases, while an inverse effect is observed for *T*. This tendency could be explained taking into account that as *M* increases and *T* decreases, DC decreases and consequently SV decreases. Similar tendency was also found for other materials such as barley and corn-bean mixtures [6, 23, 36]. Moreover, González et al. [41] have found that as the extrusion temperature increases, extrudate structure became lighter, as a consequence of the reduction of wall thickness of the pores.

It is interesting to point out that the effects of *T* on SV is higher for Fortuna than for Paso 144. This is in agreement with the results obtained by González et al. [15] which have shown that rice variety, containing lower amylose content, are more sensible to thermal effects than those of higher amylose content. 

### 3.4. Water Absorption

Water absorption values corresponding to Paso are higher than those of Fortuna. According to the ANOVA results, the lack of fit corresponding to Paso 144 was significant (*P* < 0.0396); thus the regression model does not explain adequately the response variability, suggesting that some other factor, not controlled during the experiment, played some role. 

**Figure 4:**
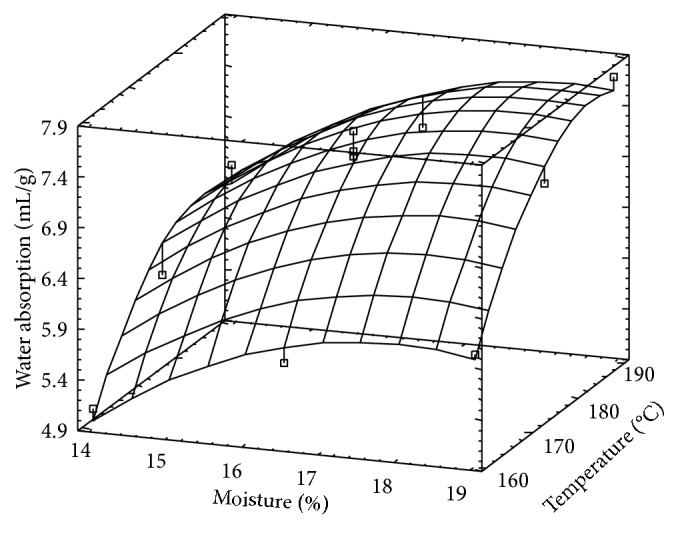
Surface response for water absorption corresponding to Fortuna rice variety.

For Fortuna rice, WA was directly related to both variables and the highest value corresponded to the sample obtained at 190°C and 19% (Figure 4). There is an important difference in the tendency observed for the moisture effect, because other responses evaluated, decreased as *M* increased. This would suggest that WA is more affected by thermal effect than that of friction. It is known that as extrusion conditions minimize friction effects, destruction of granular structure is also minimized and consequently WA is improved [18].

### 3.5. Solubility

*S* values corresponding to Paso were significantly lower than those of Fortuna, which is in agreement with the results obtained by other authors, who have found an inverse relation between amylose content and solubility [6, 8, 19, 29, 32]. Moreover, it is also important to point out that as DC increases, *S* increases as a consequence of starch granule destruction, implying a reduction of WA [23]. 

**Figure 5:**
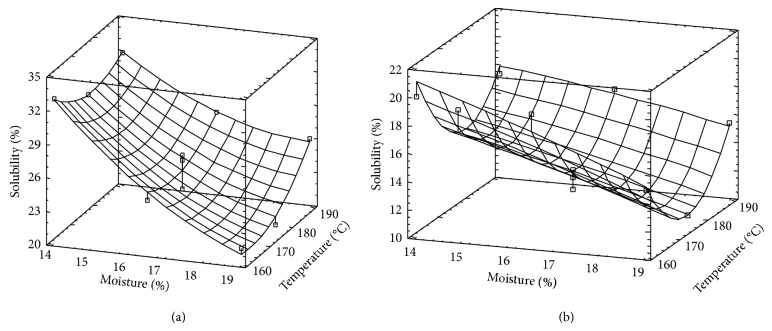
Surface response for solubility corresponding to Fortuna rice (a) and Paso 144 rice variety (b).

Solubility (*S*) response surfaces corresponding to each rice variety are observed in Figures 5(a) and 5(b). 

In the case of Fortuna, the surface is almost a plane and *S* is only affected by *M*, suggesting that friction effects are the most important ones. As it was observed by other authors [23, 42], *S* is directly related with SMEC and SV. Again, the lower amylose content of this variety could explain the high sensitivity to friction. 

In the case of Paso 144, the surface shows a curvature in *T* direction, as it is expected, since *T* ^2^ term was highly significant (*P* < 0.00112). As it was observed for WA, *S* decreases as *M* increases and similar explanation could be done regarding friction effects. The highest *S* values corresponded to samples obtained at low *M* level (14%) and at 160°C and 190°C.

The existence (at any *M* level in the range of this study) of minimum values of *S*, at intermediate *T* level (175°C), is difficult to explain. *S* results suggest that the behavior of both varieties inside the extruder would be different. On one hand, Fortuna shows much higher *S* values than Paso 144, indicating that a higher degree of cooking was reached by Fortuna rice. This is in agreement with that expected for the variety with lower amylose content. Moreover, in the extrusion of rice grits obtained by milling white rice (without the germ and the bran) and for similar extrusion conditions [15], *S* values were much higher than those obtained in this study, suggesting that lower degree of cooking is obtained when grits from whole grains is used in comparison with those coming from degermed and dehulled grains. Regarding that, Pan et al. [19] reported that the addition of oil and bran to rice grits caused a reduction of energy consumption, indicating a reduction of degree of cooking. 

Beside that, DC is, in general, directly related to *T*; in our case, it is true only at *T* higher than 175°C and for any moisture level. It seems that an increase of *T* from 160°C to 175°C produces a reduction of DC (lower *S*), so the effect of the reduction of friction caused by this increase of *T* would be higher than the thermal effect on DC. Then, above 175°C the relative importance of these two effects is inversed and the expected effect of *T* is verified. 

These results would indicate that the presence of germ (oil) and bran (fiber) could reduce the DC reached by the extrudate. This was confirmed by sample observation under microscope, which revealed the presence of native starch in all Paso samples. Even though no quantitative determination was done, the proportion of native starch present in samples corresponding to Paso 144 was much higher than that corresponding to Fortuna; moreover, no native starch was observed in Fortuna samples obtained at low-level moisture (14%). This incomplete cooking process is attributed to perturbation of the particles transport inside the extruder that not only could retard the cooking process of starchy particles caused by friction, but also could broaden the residence time distribution of particles inside the extruder. So, some particles would reach the die very fast, without suffering much change (remaining in native state). This interference on the cooking process of starch caused by the presence of oil and fiber could be overlapped by changing the extrusion condition in order to increase DC, for example, reduce die diameter, increase screw compression ratio, reduce moisture content, and reduce particle size. 

### 3.6. Dispersion Viscosity Evaluation

Since the milled extrudate could be used as a base for gruel and cream soup formulations, dispersion viscosity becomes an important factor to be taken into account.

The evaluation of dispersion viscosity was done on the samples obtained at 14% and 160°C, since these extrusion conditions permitted the highest DC. Viscosity was obtained from rheograms at 135 s^−1^ shear rate according to Pérez et al. [3] since this level of shear rate would be in range of that exerted inside the mouth when a fluid food is ingested.  Table 4 shows the results of extrudate flour viscosity evaluation: power law parameters (*k* and *n*) and regression coefficient obtained from the rheograms made for each solid concentration (8% and 11%) and for each rice varieties. 

It is observed that power law model adjusted adequately the rheograms data (*r* > 0.994). Dispersion prepared at 11% solids concentration showed plastic behavior (yield stress). Dispersion corresponding to Fortuna variety showed lower viscosity than Paso, indicating that samples having lower degree of cooking give thicker dispersion. Beside that, a direct correlation was observed between water absorption and viscosity (*r* > 0.945), as it was expected, since water absorption is an indicator of dispersion thickness. These results are in agreement with those obtained in other works [3, 43].

## 4. Conclusions

Physical characteristics of extrudates depend on extrusion conditions and rice variety used. Low amylose variety showed values of SMEC, SV, and *S* higher than those of higher content; on the contrary, WA was lower, indicating that the last one reached lower degree of cooking. Both varieties showed similar values of expansion rate. 

Regarding the extrusion conditions, 14% *M* and 175°C could be selected to obtain a good expanded product, with high expansion and intermediate degree of cooking.

Since degrees of cooking reached by both rice samples (under the same extrusion condition) were lower than those obtained using grits from polish rice, it is suggested that the presence of germ and bran interferes the cooking process by both decreasing friction level and by broadening residence time distribution. 

The higher protein, fiber, and mineral content of brown rice in comparison to polish rice implies nutritional advantages, so the use of brown rice to produce snacks products is highly recommended.

## Figures and Tables

**Table 1:** Composition of raw rice∗∗.

Sample	Proteins∗ (g kg^−1^)	Ash∗ (g kg^−1^)	Moisture (g kg^−1^)	Lipids (ether extract)∗ (g kg^−1^)	Total Dietary Fibre∗ (g kg^−1^)
Fortuna	71.5 ± 2.0	11.8 ± 0.4	118.6 ± 1.2	19.8 ± 0.3	52.7 ± 1.7
Paso	75.9 ± 0.9	15.0 ± 0.1	115.7 ± 0.7	23.6 ± 0.4	55.3 ± 1.7

^*^Dry base; ∗∗average ± SD.

**Table 2:** Specific mechanical energy consumption (SMEC), expansion (*E*), specific volume (SV), water absorption (WA), and solubility (*S*%) corresponding to the eleven experimental extruded samples and for the two rice varieties.

Extrusion conditions		SMEC (J/g)		*E*		SV (mL/g)		WA (mL/g)		*S*%	
*M* (%)	*T* (°C)	*F*	*P*	*F*	*P*	*F*	*P*	*F*	*P*	*F*	*P*
**14**	**160**	703	637	3.36	3.46	5.39	7.26	5.1	6.6	33.0	20.1
**14**	**175**	671	590	3.38	3.50	6.52	8.27	5.9	8.4	30.8	17.0
**14**	**190**	620	493	3.36	3.42	8.64	7.89	6,5	7.9	31.9	17.5
**16.5**	**160**	609	585	3.60	3.40	5.43	6.12	5.7	6.9	24.9	19.5
**16.5**	**175**	547	575	3.14	3.01	5.22	6.65	7.5	7.5	23.4	13.5
**16.5**	**175**	534	543	3.03	3.16	5.65	6.98	7.3	7.3	23.3	12.1
**16.5**	**175**	541	566	3.12	3.17	5.29	6.90	7.2	7.22	25.8	12.9
**16.5**	**190**	541	483	2.89	2.95	7.23	7.83	7.0	8.3	27.5	17.2
**19**	**160**	483	511	2.55	2.92	3.47	4.53	6.0	7.6	21.5	14.8
**19**	**175**	462	501	2.77	2.76	4.29	5.39	7.2	7.0	21.0	11.1
**19**	**190**	409	407	2.46	2.18	5.94	5.84	7.7	8.1	26.1	15.5

*M*: moisture; *T*: temperature; *F*: Fortuna rice variety; *P*: Paso 144 rice variety.

**Table 3:** Degrees of significance (*P* value) of each term of the regression model (source of variation), corresponding to each response and for the two rice varieties.

Source of variation	SMEC		*E*		SV		WA		*S*%	
	*f*	*p*	*f*	*p*	*f*	*f*	*f*	*p*	*f*	*p*
*M*	**0.0006**	**0.0174**	**0.0043**	**0.0043**	**0.0067**	**0.0030**	**0.0043**	0.6779	**0.0204**	**0.0169**
*T*:	**0.0048**	**0.0129**	**0.0351**	**0.0351**	**0.0055**	**0.0129**	**0.0351**	**0.0128**	0.2554	0.1337
*M* ^2^	0.7563	0.2007	**0.0448**	**0.0448**	0.2361	0.0968	**0.0448**	0.2160	0.3121	0.7205
*M* × *T*	0.5381	0.3411	0.5534	0.5534	0.2331	0.1886	0.5534	0.1270	0.2171	0.1510
*T* ^2^	0.1403	0.0978	0.9086	0.9086	**0.0415**	0.2467	0.9086	0.8320	0.2432	**0.0112**
Lack of Fit	0.0794	0.7997	0.0515	0.0515	0.2398	0.1593	0.0515	**0.0396**	0.8966	0.1951

*M*: moisture; *T*: temperature; *F*: Fortuna rice variety; *P*: Paso 144 rice variety; specific mechanical energy consumption (SMEC); expansion (E); specific volume (SV); water absorption (WA); and solubility (S%).

**Table 4:** Power law parameters (*k* and *n*), regression coefficient (*r*), and viscosity at 135 s^−1^ (Pa·s^−1^), corresponding to flour dispersion at 8% and 11% solid concentration and for each rice varieties.

Sample	W/W %	*τ*_0_ (Pa)	*k*	*n*	*r*	*η* 135 s^−1^(Pa·s^−1^)
Fortuna	8	0	0,436	0,497	0,9948	0,037
	11,0	4,5	1,053	0,636	0,9999	0,210
Paso	8	0	0,686	0,433	0,9994	0,042
	11,0	7,2	3,670	0,550	0,9988	0,451
